# Micro-photoluminescence spectroscopy of detonation nanodiamonds containing germanium-vacancy centres

**DOI:** 10.1039/d5na00795j

**Published:** 2025-10-24

**Authors:** Yoshiki Saito, Yushi Okuda, Yoshihiro Tomoi, Taiki Shimamura, Takuya Matsuda, Yuto Makino, Masaaki Ashida

**Affiliations:** a Graduate School of Engineering Science, The University of Osaka 1-3 Machikaneyama-cho Toyonaka Osaka 560-8531 Japan ashida@mp.es.osaka-u.ac.jp; b Daicel Corporation 1239 Shinzaike, Aboshi-ku Himeji Hyogo 671-1283 Japan

## Abstract

A germanium-vacancy (GeV) centre in nanodiamond is a promising candidate for bright single-photon emitters, fluorescence labelling and quantum sensing due to its narrow zero-phonon line (ZPL) and large Debye–Waller factor. Recently, the GeV centre-containing nanodiamond (GeV-ND) was fabricated by a detonation process, which provides a scalable method capable of producing large quantities of ND particles. However, the optical properties of the GeV-ND at the single-particle level have not been well studied. In this study, we conducted spatially resolved photoluminescence (PL) mapping on sparsely distributed GeV-ND particles to examine luminescence characteristics across individual particles. The GeV-ND exhibited a narrow ZPL accompanied with a broad emission band. By comparing with well-characterized PL spectra of nitrogen-vacancy (NV) centres in detonation NDs reported in previous single-particle-level studies for magnetometry sensing applications, we attribute the broad background to luminescence from the NV centres coexisting with the GeV centres in NDs. By subtracting this background, we identified the pure GeV emission. Some GeV-NDs showed ZPL linewidths as narrow as 28 meV at room temperature, suggesting the presence of optically promising GeV centres. These findings clarify the origins of PL observed in GeV-NDs and demonstrate their potential as scalable quantum emitters.

## Abstract 2

Micro-photoluminescence spectroscopy revealed the origin of background emission in detonation-synthesised GeV nanodiamonds, and showed zero-phonon line emission as narrow as 28 meV at room temperature.

## Graphical abstract

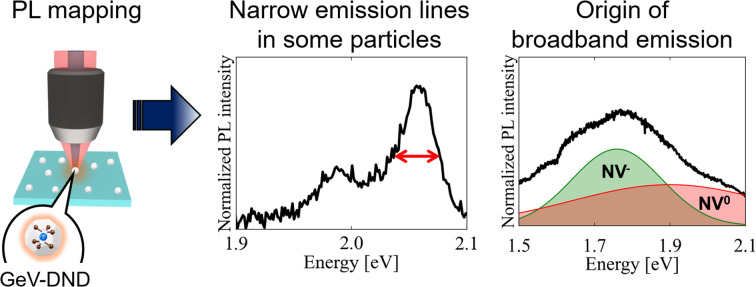

## 1. Introduction

Nanodiamonds containing colour centres have attracted increasing attention in the fields of quantum technologies and biomedical sciences, such as temperature sensing and bioimaging, owing to their excellent optical and spin properties as well as their inherent biocompatibility.^1–3^ Among various types of colour centres, the nitrogen-vacancy (NV) centre in diamond possessing *C*_3v_ symmetry has been widely studied owing to its spin coherence properties, room-temperature optical addressability, and stable emission.^4–8^ However, they also show strong electron–phonon coupling, which leads to a weak zero-phonon line (ZPL) and a dominant phonon sideband (PSB) in their photoluminescence (PL) spectra. The negatively charged NV (NV^−^) centre has a ZPL at 1.95 eV (637 nm), with a broad PSB from 1.55 to 1.91 eV (650–800 nm), resulting in a Debye–Waller factor (DWF) of approximately 4%,^4,9–11^ where the DWF is defined as the ratio of the ZPL intensity to the total emission intensity. The neutral NV (NV^0^) centre shows a ZPL at 2.16 eV (575 nm) with a broad PSB from 1.75–2.21 eV (560–710 nm).^4,9–11^ These small DWFs have been considered limiting factors for applications requiring emission dominated by the ZPL only, such as indistinguishable single-photon generation or high-contrast fluorescence imaging.^9,12,13^ In contrast, a germanium-vacancy (GeV) centre, has emerged as promising alternatives to NV centres due to their significantly reduced electron–phonon coupling. GeV centres possess a split-vacancy configuration with *D*_3d_ symmetry, which significantly suppresses electron–phonon coupling.^9,14–17^ The GeV centres exhibit a narrow ZPL at 2.06 eV with a linewidth of 9–30 meV and a DWF of 0.3–0.6, and these properties make nanodiamonds containing GeV centres (GeV-NDs) suitable for bright single-photon emitters, fluorescence labelling and quantum sensing.^1,12,18–26^

To enable broader application, the synthesis of GeV-NDs has been actively studied in recent years.^20–22^ Recently, the GeV-NDs synthesised by high-pressure high-temperature (HPHT) and chemical vapour deposition (CVD) methods have recently been reported.^20–25^ In HPHT-grown GeV-NDs with particle sizes of approximately 20 nm, room-temperature ZPL linewidths as narrow as 17 meV and Debye–Waller factors (DWFs) up to ∼0.6 have been achieved.^24^ CVD-grown GeV-NDs, typically ranging in size from several tens to hundreds of nanometres, have also been reported, with ZPL linewidths of ∼30 meV^25^ or even up to ∼50 meV in some cases.^20,25^ These linewidths are still considered optically high-quality and suitable for many practical quantum photonic applications. However, these synthetic methods are generally limited to the laboratory scale. Here, a detonation synthesis technique is widely known to provide NDs with a particle size of less than 10 nm on a practical scale.^27,28^ This process can yield particles as small as ∼3 nm,^27^ while also producing larger ones in the range of 20–30 nm.^32^ The detonation synthesis is commenced by a detonation of a solid explosive with a negative oxygen balance. Carbon atoms of organo-nitro-explosive molecules are momentarily converted into NDs under the high-pressure high-temperature detonation wave propagating through the solid explosive. Recently, we have demonstrated a direct synthesis of GeV-NDs through a detonation technique (GeV-DNDs), which employed the explosive containing an aromatic Ge compound as a Ge source.^29^ GeV-DNDs have already been evaluated for temperature sensing in the 295–315 K range,^30^ with performance reported to be comparable to that of bulk diamond, underscoring their suitability for nanoscale measurements. In addition, their sizes are advantageous for biomarkers for imaging down to the single-protein level, as nanoparticles ideally should match biomolecular dimensions.^31^ These considerations suggest that GeV-DNDs could be particularly promising as nanoscale temperature sensors, nanoscale fluorescent markers, and potential single-photon emitters.

In our previous study, ensemble PL measurements were performed on the GeV-DND samples drop-cast at a high concentration (10 wt%) onto glass substrates.^32^ These measurements confirmed strong GeV emission at room temperature. Its ZPL exhibited an average full width at half maximum (FWHM) of 59 meV. Compared to GeV-NDs synthesised *via* HPHT or CVD methods, our detonation-synthesised samples showed broader linewidths and lower DWFs. Furthermore, a broad background luminescence component overlapping the GeV ZPL—likely arising from NV centres and surface defects—was observed, but its origin remained unresolved. Because these measurements were performed on ensembles, the observed linewidth likely reflected inhomogeneous broadening arising from particle-to-particle variability, making it unclear whether any individual GeV-DND might reach the optical properties of the best HPHT/CVD-grown emitters. Resolving these issues is therefore essential for evaluating the true quantum-emitter potential of GeV-DNDs.

In this study, we addressed these issues by conducting spatially resolved PL mapping on sparsely dispersed GeV-DNDs. Through this experiment, we identified that the background luminescence arises from NV^0/−^ centres and surface defects. The histogram of ZPL linewidths revealed that the majority of GeV-DNDs exhibit linewidths centred around 40 meV. This value is comparable to those reported in previous studies demonstrating temperature sensing using GeV centres, including those fabricated *via* ion implantation into CVD diamond.^26^ Furthermore, a small fraction of particles exhibited exceptionally narrow linewidths down to 28 meV, which is consistent with the optical properties expected of single-photon emitters.

## 2. Experimental methods

The GeV-DND particles used in this study were the same samples as those reported previously.^32^ Morphological and structural characterisations (transmission electron microscopy (TEM) and X-ray diffraction (XRD)) of the GeV-DNDs have already been reported.^32^ In brief, the procedure involves: (i) detonation of a mixed explosive (2,4,6-trinitrotoluene (TNT), 1,3,5-trinitro-1,3,5-triazacyclohexane (RDX), and tetraphenylgermane (TPG)) under a CO_2_ atmosphere; (ii) purification of the detonation products by acid treatment, alkali treatment, and subsequent air oxidation. The GeV-DNDs were mixed with water to prepare a 0.02 wt% sample. Compared to our previous study,^29^ the concentration was reduced by a factor of 500 to allow the optical properties of individual nanodiamond particles to be resolved. The diluted suspension was drop-cast onto glass substrates and dried under ambient conditions to prepare samples for measurement.

The confocal micro-PL mapping measurements were performed on the drop-cast sample. A continuous wave (CW) laser was used at 2.33 eV. A 100× objective lens (NA = 0.9) was used. A CCD camera was employed as the detector, with a 30 cm spectrometer and a diffraction grating of 150 gr. mm^−1^. The mapping measurements were conducted using a piezoelectric automated stage integrated into the microscope system. The mapping range was set to 150 μm × 150 μm with a step size of 1 μm. The PL spectra at each measurement point were acquired with an exposure time of 1 second, resulting in a total of 22 500 measurement data points. This measurement allows for the comprehensive characterization of the optical properties of individual nanodiamond particles.

## 3. Results and discussion

### 3.1 PL spectral characteristics of low-GeV-emission regions

**Fig. 1:**
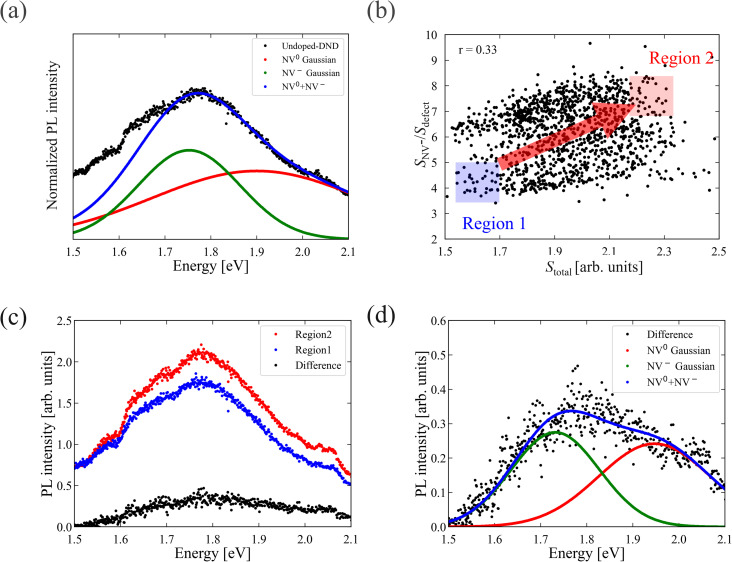
(a) Representative PL spectrum (black dots) from low-GeV-emission regions. (b) Scatter plot showing the relationship between the total integrated PL intensity (*S*_total_) and the ratio of NV^−^ emission to the unidentified residual component (*S*_NV^−^_/*S*_defect_) at each measurement point. A positive correlation (*r* = 0.33) is observed. The arrow in the plot is provided as a visual guide. (c) Representative PL spectra selected from two representative regions: region 1 (blue), with low *S*_total_ and low *S*_NV^−^_/*S*_defect_; and region 2 (red), with high values in both metrics. (d) The difference spectrum (black dots) between region 1 and 2 is shown, along with Gaussian fits to the NV^−^ (green) and NV^0^ (red) components. The blue dots represent the sum of the fitted components.

To investigate the broad background luminescence component overlapping the GeV ZPL (2.06 eV) and discuss the origin, we chose some GeV-DND samples that showed only weak or no detectable GeV centre emission. The detailed selection method for these low-GeV-emission samples is described in the SI. Black dots in Fig. 1(a) show a representative PL spectrum. The broadband emission was observed in the range of 1.50–2.10 eV. This emission overlapped with the energy regions associated with NV^0^ centres (1.90–2.15 eV) and NV^−^ centres (1.65–1.97 eV).^4,9–11^

In DNDs, nitrogen atoms from the explosive molecules are known to be spontaneously incorporated into the diamond lattice, resulting in the NV centre formation naturally.^33,34^ In many cases, it is difficult to discern the zero-phonon lines (ZPLs) of NV centres in such ultrasmall nanodiamonds.^33,35–39^ These previous studies suggest that the broad background emission originates from NV centre-related PSBs.

To verify this, we fitted the spectrum using Gaussian functions: a lower-energy component (NV^−^ Gaussian, green) and a higher-energy component (NV^0^ Gaussian, red). Because variability in ZPL features further reduces reliability, we focus on our analysis to the PSB region and adopted this simplified two-Gaussian model. The fit yielded an NV^−^ Gaussian peak at 1.76 eV (FWHM 277 meV) and an NV^0^ Gaussian peak at 1.87 eV (FWHM 644 meV). Although the fitted peak positions differ from those of NV centres in bulk diamond (1.82 eV with FWHM ∼280 meV for NV^−^ and 1.98 eV with FWHM ∼350 meV for NV^0^),^11^ the obtained values are essentially identical to those extracted from spectral data for NV centres in DNDs,^33^ where we found NV^−^ at 1.77 eV (FWHM ∼280 meV) and NV^0^ at 1.90 eV (FWHM ∼500 meV), reflecting the enhanced electron–phonon coupling and the influence of surface effects characteristic of ultrasmall nanodiamonds. Surface effects, including spectral shifts due to surface termination and local environment, have been reported in prior studies.^36^

We further found that the NV^−^/NV^0^ emission ratio in our samples was 0.75, which closely matches the previously reported value of 0.86 for NV centre-containing DNDs.^33^ This consistency, along with the spectral fitting results, indicates that the observed broad emission originates primarily from NV centres. Together, our fitting and these literature precedents strongly support attributing the broad background emission to NV centres.

However, a residual component around 1.55 eV remains, which cannot be explained by NV centres alone and is likely due to surface defects inherent to DNDs. Indeed, surface-defect emission in 5 nm nanodiamonds has been reported at energies below 1.65 eV.^40^

To examine the relationship between the intensity of background emission and the relative contribution of NV^−^ centres, we plot a scatter diagram as shown in Fig. 1(b). The horizontal axis represents the total PL intensity *S*_total_. The vertical axis represents the ratio of the NV^−^ emission intensity to the background component *S*_NV^−^_/*S*_defect_. Here, *S*_NV^−^_ corresponds to the integrated area of the Gaussian function fitted to the NV^−^ component, and *S*_defect_ is defined as the integrated PL intensity over the 1.50–1.65 eV range in the residual spectrum after subtracting both the NV^−^ and NV^0^ components from the original spectrum. The NV^0^ component was not included in this ratio because its PSB (1.90–2.15 eV) partially overlaps with the GeV ZPL, making it difficult to extract independently. A positive correlation (*r* = 0.33) was observed *S*_total_ and *S*_NV^−^_/*S*_defect_. Correlations were quantified using the Pearson correlation coefficient *r*_xy_ (see SI). From this scatter plot, we selected two typical normalized spectra: one from region 1 (blue-shaded area in Fig. 1(b)), where both *S*_total_ and *S*_NV^−^_/*S*_defect_ are low, and the other from region 2 (red-shaded area in Fig. 1(b)), where both values are high. These representative PL spectra for regions 1 and 2 are shown in Fig. 1(c) (blue and red dots, respectively). Additionally, we took the difference between the two spectra (black dots). Fig. 1(d) shows the result of fitting the difference spectrum (blue dots) with a sum of Gaussian functions. These results indicate that the intensity of the emission is strongly dependent on the number of NV^−^ and NV^0^ centres present in individual DND particles. These findings provide a crucial reference for interpreting the background emission observed in GeV-DNDs, as discussed in the following section.

### 3.2 PL spectral characteristics of GeV centres

**Fig. 2:**
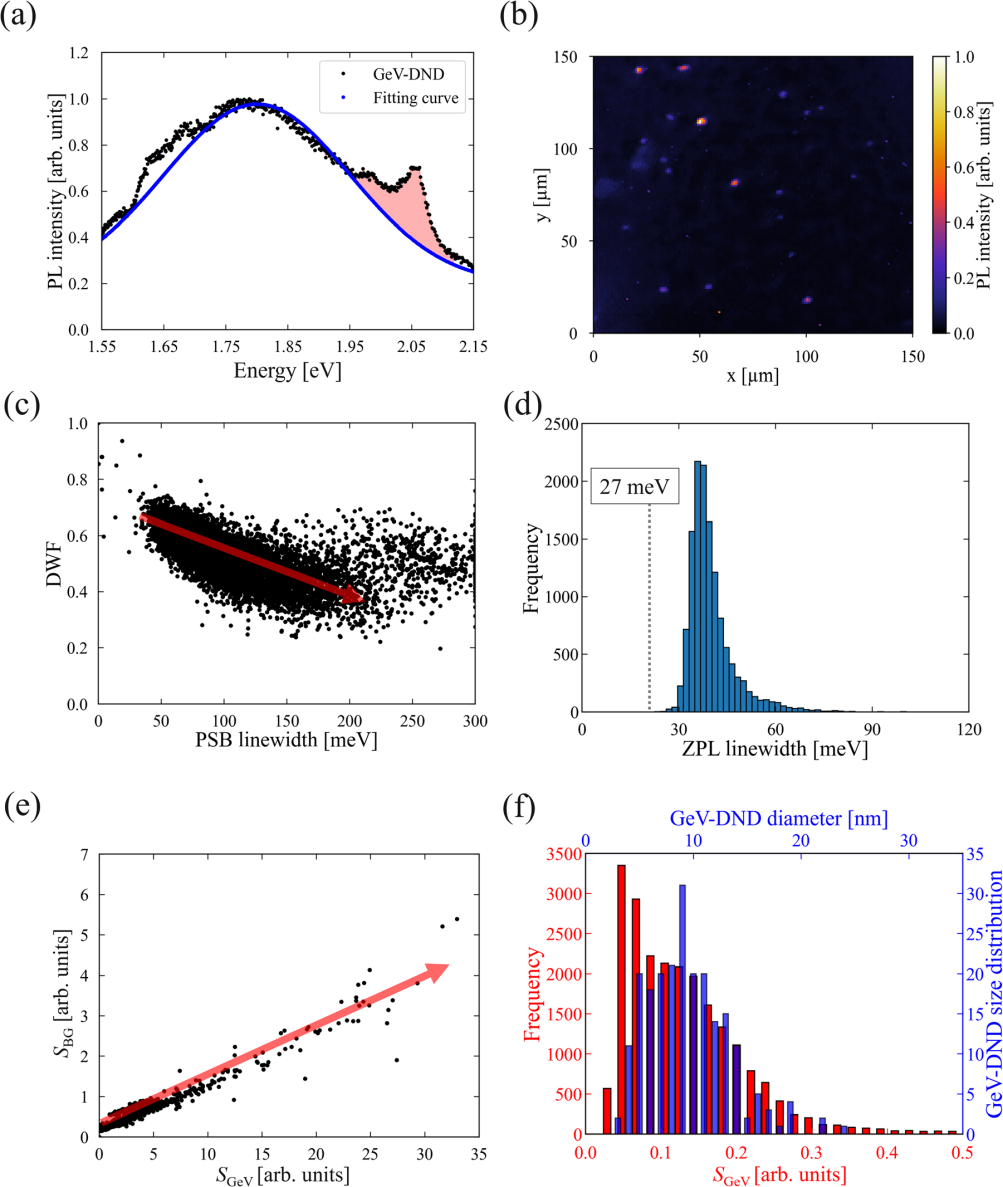
(a) Representative PL spectrum (black dots) from a GeV-emitting region, overlaid with a fitted background spectrum (blue line) based on a two-Gaussian model for NV-related emission. (b) PL intensity map obtained from spatially-resolved measurements; colour scale represents the integrated intensity in the GeV emission range. (c) Correlation between the Debye–Waller factor (DWF = *S*_ZPL_/(*S*_ZPL_ + *S*_PSB_)) and the linewidth of the phonon sideband (PSB), obtained from peak fitting of GeV center emission. The arrow in the plot is provided as a visual guide. (d) Histogram of ZPL linewidths, showing a distribution with a minimum of 28 meV. (e) Scatter plot of *S*_GeV_, the integrated PL intensity of the GeV center (1.90–2.10 eV), *versus S*_BG_, the integrated background PL intensity in the same energy range. The arrow in the plot is provided as a visual guide. (f) Comparison between the histogram of *S*_GeV_ and the previously reported particle size distribution of GeV-DNDs (from ref. 29).

Fig. 2(a) shows a representative PL spectrum obtained from a region exhibiting GeV centre emission in the GeV-DND sample (black dots), featuring the GeV ZPL at 2.06 eV and PSB at 1.98 eV. These peaks are superimposed on background emission components. To extract the emission component from the GeV centre, we applied the same two-Gaussian fitting used in Section 3.1 to model the background. The fitting yielded two broad background components with central energies at 1.96 eV and 1.73 eV and linewidths of 352 meV and 310 meV, respectively. This strong agreement supports that the background emission in GeV-emitting regions shares the same origin as that in the low-GeV-emission regions, namely NV centres and surface-related luminescence. Moreover, as described in “PL Spectral Characteristics of low-GeV-emission regions”, a residual emission component around 1.55 eV remains after subtracting the two-Gaussian fit. This residual cannot be accounted for by GeV or NV centre alone and is likely attributable to surface defects inherently present in DNDs.^40^

Fig. 2(b) shows the spatial PL intensity map of GeV emission. The sparsely distributed bright spots correspond to individually dispersed GeV-DNDs on the substrate. We analysed individual GeV-DNDs by applying two-peak fitting to background-subtracted spectra (see SI). In this fitting, the higher-energy peak and the lower-energy peak were treated as the ZPL and PSB, respectively. We extracted the ZPL and PSB linewidths and evaluated their correlation with the DWF. In this study, DWF was defined as DWF = *S*_ZPL_/(*S*_ZPL_ + *S*_PSB_), where *S*_ZPL_ and *S*_PSB_ are the integrated intensities obtained from a simultaneous fit to the ZPL and PSB. The PSB linewidth was taken as FWHM of the fitted PSB peak (see SI). Fig. 2(c) shows a negative correlation (*r* = −0.73), suggesting that stronger electron–phonon coupling leads to broader PSB emission and reduced DWF in ∼10 nm nanodiamonds.

Fig. 2(d) shows a histogram of ZPL linewidths for 22 500 measurement spots. The distribution is centred around 40 meV, with an average linewidth of 44.2 meV, a median of 39.0 meV, and an interquartile range of 35.9–46.6 meV, indicating that most particles exhibit linewidths near this value. While a small fraction exhibited broader linewidths up to ∼60 meV, a limited number of particles exhibit particularly narrow linewidths down to 28 meV.

The predominance of ∼40 meV linewidths observed in ∼10 nm GeV-DNDs is thought to result from structural factors such as enhanced electron–phonon coupling, local lattice strain, and surface-related perturbations, which are characteristic of detonation-synthesised nanodiamonds. Although these linewidths are broader than those of the highest-quality GeV centres, used in bulk-diamond GeV centre thermometry, which has achieved 0.1 K temperature resolution over the 150–400 K range,^26^ and are therefore considered sufficiently narrow for practical use.

We especially found that some GeV-DNDs—despite their ultrasmall size of approximately 10 nm—exhibited ZPL linewidths as narrow as 28 meV. This value is comparable to those reported for HPHT-synthesised GeV-NDs of 20 nm^21^ and even for CVD-grown samples around >100 nm,^22^ demonstrating that high optical quality can be achieved even in DND. This value is comparable to that reported for GeV centres (27 meV) exhibiting antibunching behaviour.^19^ Furthermore, antibunching has been reported in directly detonation-synthesised SnV-DNDs.^41^ Considering the ultrasmall particle size of ∼10 nm, incorporation of multiple GeV centres within a single DND is unlikely to be stable. In fact, Bolshakov *et al.* reported that for SiV centres in CVD-grown diamond, the PL intensity peaked at moderate doping levels around particle sizes of ∼20 nm and rapidly quenched at higher concentrations, where the average inter-centre distance becomes too small.^42^ This behaviour was attributed to defect formation and aggregation of colour centres, both of which hinder stable optical activity. By analogy, in ultrasmall DNDs, the coexistence of more than a few GeV centres is expected to be unfavourable. Taken together, these parallels strongly suggest that GeV centres in DNDs are also likely to exist as single or very few emitters.

In our previous ensemble measurement of GeV-DNDs, the average ZPL linewidth was reported as 59 meV.^32^ This discrepancy likely arises from differences in background subtraction methods: the previous study used spectral subtraction with PL spectrum of undoped-DNDs, whereas the present work employed Gaussian fitting of NV^0^ and NV^−^ components. Such methodological differences should be considered when comparing the two results.

Fig. 2(e) shows a scatter plot of GeV centre integrated PL intensity (*S*_GeV_), defined as the background-subtracted spectral area in the 1.90–2.10 eV range, *versus* the background integrated PL intensity (*S*_BG_) in the same energy window, calculated from the fitted sum of NV^0/−^ related Gaussian components. A very strong positive correlation with a correlation coefficient of 0.96 was observed, indicating a systematic relationship between GeV centre emission and background emission components.

To further investigate this relationship, Fig. 2(f) compares the histogram of GeV centre PL intensities (*S*_GeV_) with the particle size distribution of GeV-DNDs reported previously.^32^ Both distributions exhibit a skewed profile with long tails toward higher values—higher PL intensities for GeV emission and larger diameters for particle size. This similarity suggests that the strong correlation observed in Fig. 2(e) likely reflects a particle-size-dependent incorporation of Ge and N during detonation synthesis. Larger nanodiamonds may accommodate more GeV and NV centres, contributing to stronger emission in both channels.

However, this overall trend does not exclude the presence of NDs containing only a single GeV centre. In fact, the observation of extremely narrow ZPL linewidths down to 28 meV strongly suggests that some particles exhibit emission characteristics comparable to those of single-photon emitters. These results imply that both multi-centre and single-emitter-like particles coexist in the sample. The present comprehensive analysis reveals the distribution characteristics, while also showing that high-quality GeV centres suitable for quantum applications can be individually identified within the ensemble.

## 4. Conclusion

In summary, to investigate their optical properties at the individual particle level, we conducted spatially resolved confocal micro-PL mapping on GeV-DNDs. We observed broadband luminescence, suggesting that the background emission originates from the NV^0^ centre, the NV^−^ centre, and surface defects.

The histogram of ZPL linewidths revealed that the majority of GeV-DNDs exhibit linewidths centred around ∼40 meV. This value is comparable to those reported for GeV centres in NDs synthesised by CVD, indicating that detonation-synthesised particles can reach an optical quality similar to that achieved by conventional methods.^20^ In addition, a small fraction of GeV-DNDs showed exceptionally narrow ZPL linewidths down to 28 meV. This linewidth is comparable to that of single photon emitters,^19^ suggesting the potential application of detonation-synthesised GeV-DNDs as high sensitive quantum sensors.

These findings advance our understanding of the optical properties of GeV centre-containing NDs synthesised by the detonation process.

## Conflicts of interest

There are no conflicts to declare.

## Supplementary Material

NA-OLF-D5NA00795J-s001

## Data Availability

The data that support the findings of this study are available from the corresponding author upon reasonable request. Supplementary information is available. See DOI: https://doi.org/10.1039/d5na00795j.
