# Supplementary material for: Micro-photoluminescence spectroscopy of detonation nanodiamonds containing germanium-vacancy centres
Source: Nanoscale Adv. 2025 Oct 24;7(24):7943–9. doi: 10.1039/d5na00795j (PMC12550598; doi:10.1039/d5na00795j)
Supplement: NA-007-D5NA00795J-s001 [file NA-007-D5NA00795J-s001.pdf]

Supplementary Information (SI) for Nanoscale Advances.
This journal is © The Royal Society of Chemistry 2025

Micro-photoluminescence Spectroscopy of Detonation Nanodiamonds Containing Germanium-Vacancy Centres

Yoshiki Saito, Yushi Okuda, Yoshihiro Tomoi, Taiki Shimamura, Takuya Matsuda, Yuto Makino and Masaaki Ashida*

Supporting information

Experiment

Definition of low-GeV-emission regions

To identify measurement spots with negligible GeV centres emission (low-GeV-emission regions), we applied a specific fitting and integration procedure to each PL spectrum obtained from the GeV-DND sample.

First, we fitted the spectrum using two Gaussian functions representing the NV^0 and NV^- centre emissions. The fitting was performed in two side regions: 1.75–1.85 eV and 2.12–2.17 eV, where the contribution from GeV centres is minimal. The centre positions and linewidths of the Gaussian functions were fixed as described above, and only the amplitudes were treated as fitting parameters.

After determining the background emission by this fitting, the GeV centre emission intensity was defined as the integrated PL intensity over the 1.95–2.10 eV range after subtracting the fitted NV^0 and NV^- components.

Measurement spots with an extracted GeV emission intensity of 200,000 counts or less were classified as low-GeV-emission regions for further analysis.

Comparison of Narrow-Linewidth GeV Emission with Ensemble-Averaged Spectrum

To highlight the identification of GeV centres with exceptionally narrow zero-phonon line (ZPL) emission, we present a comparison between two representative PL spectra in Fig. S1. One spectrum was acquired from a localised spot in the present spatially resolved PL mapping, showing a ZPL linewidth of 28 meV, the narrowest observed in this study. The other spectrum corresponds to the ensemble-averaged PL spectrum of GeV-DNDs previously reported, which exhibited a broader ZPL linewidth of 59 meV. This comparison highlights how spatially resolved PL mapping reduces the effects of ensemble-level inhomogeneities and enables more localised spectral characterization.

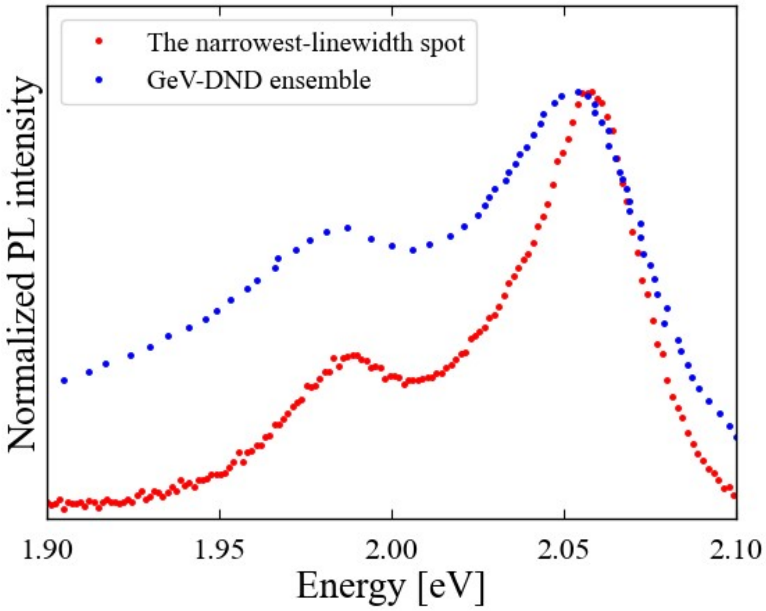

Figure S1.

Comparison of PL spectra from the narrowest-linewidth spot (28 meV) identified in this study (red) and an ensemble-averaged GeV-DND sample reported previously (blue). Both spectra are normalized and offset for clarity.

Comparison of Lorentzian and Voigt function fitting for GeV-DND PL spectra

In this study, we evaluated the appropriate line shape model for photoluminescence (PL) spectra obtained from GeV centre-containing detonation nanodiamonds (GeV-DNDs). The choice of fitting function is critical for accurately extracting spectral parameters such as ZPL linewidth and centre energy, which are essential for assessing the optical quality of GeV centres.

To compare models, we selected a representative PL spectrum acquired from a specific spot corresponding to a single GeV-DND particle. Two different fitting models were applied to this spectrum:

- A sum of two Lorentzian functions
- A pseudo-Voigt model, defined as a linear combination of a Lorentzian and a Gaussian function

The Voigt function is defined as the convolution of a Gaussian function, representing inhomogeneous broadening (e.g., strain, spectral diffusion), and a Lorentzian function, which accounts for homogeneous broadening. In contrast, the Lorentzian model assumes that the broadening is purely homogeneous. In this procedure, the amplitude, centre energy, and linewidth of both peaks are treated as free parameters; no parameters are fixed a priori, and no hard bounds are imposed beyond physically reasonable initial guesses.

Figure S2 shows the measured PL spectrum (black dots) along with the two fitting results: the red solid line represents the sum of two Lorentzian functions, and the blue dotted line represents the pseudo-Voigt model fit. Fitting was performed using the least-squares method, with amplitude, centre energy, and linewidth treated as free parameters. The fitting accuracy was evaluated based on the residual (root-mean-square error, RMSE). The two-Lorentzian model yielded a residual of 4.45×10^{-2}, while the pseudo-Voigt model gave a higher residual of 4.85×10^{-2}. This quantitative difference suggests that the two-Lorentzian model more accurately reproduces the GeV-DND PL spectrum.

Based on these results, we conclude that the PL spectrum of GeV-DNDs is best modeled using a sum of Lorentzian functions. Accordingly, all spectral fittings and analyses presented in the main text were performed using the Lorentzian model.

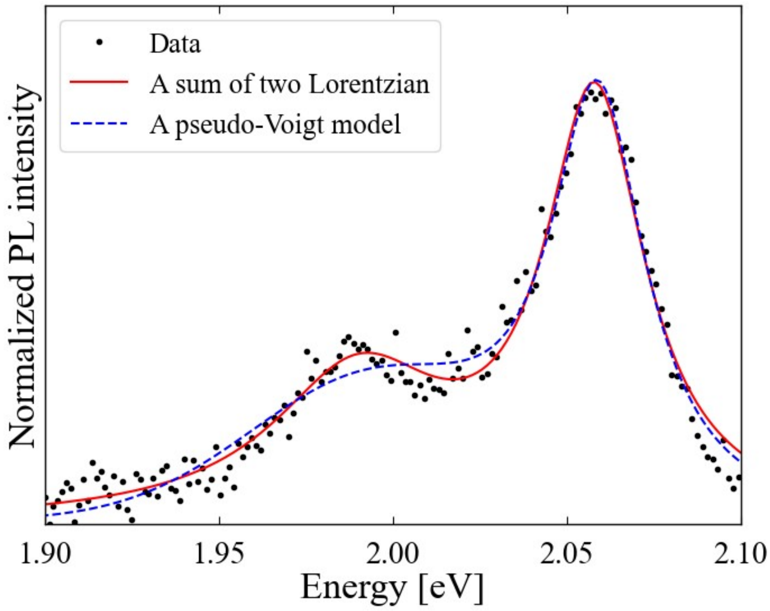

Figure S2.

Representative photoluminescence (PL) spectrum (black dots) obtained from a single GeV-DND particle, fitted using two different models. The red solid line represents a sum of two Lorentzian functions, and the blue dotted line represents a pseudo-Voigt model (a linear combination of Lorentzian and Gaussian functions). The Lorentzian model yields a smaller residual (RMSE = 4.45×10^{-2}) compared to the pseudo-Voigt model (RMSE = 4.85×10^{-2}), indicating a better fit to the experimental data.

Evaluation of Debye–Waller factor

To extract ZPL and PSB contributions from GeV-DND PL spectra, we model each spectrum as a sum of two Lorentzian functions and perform nonlinear least-squares fitting. The higher-energy Lorentzian captures the GeV ZPL, while the lower-energy Lorentzian accounts for the GeV PSB.

From the fitted peak areas, we define the integrated ZPL and PSB intensities, S_{ZPL}, S_{PSB}, as the areas of the high-energy and low-energy Lorentzian functions, respectively. The Debye–Waller factor (DWF) is then evaluated as

$$DWF = \frac{S_{ZPL}}{S_{ZPL} + S_{PSB}}$$

Evaluation of the correlation coefficient

The correlations between the Debye–Waller factor (DWF) and the PSB linewidth (FWHM) were quantified using the Pearson correlation coefficient r_{xy}, defined as

$$r_{xy} = \frac{\sum_{i=1}^{N}(x_i - \bar{x})(y_i - \bar{y})}{\sqrt{\sum_{i=1}^{N}(x_i - \bar{x})^2}\sqrt{\sum_{i=1}^{N}(y_i - \bar{y})^2}}$$

where x_i and y_i denote the paired data values, $\bar{x}$ and $\bar{y}$ are their mean values, and N is the number of samples.
